# Spatiotemporal analyses of foot and mouth disease outbreaks in cattle farms in Chiang Mai and Lamphun, Thailand

**DOI:** 10.1186/s12917-020-02392-6

**Published:** 2020-06-01

**Authors:** Orapun Arjkumpa, Chalutwan Sansamur, Pakdee Sutthipankul, Chaidate Inchaisri, Kannika Na Lampang, Arisara Charoenpanyanet, Veerasak Punyapornwithaya

**Affiliations:** 1 Faculty of Veterinary Medicine, Chiang Mai University, Chiang Mai, Thailand; 2 Lamphun Provincial Livestock Office, Lamphun, Thailand; 3 Veterinary Epidemiology and Economics Group, Faculty of Veterinary Medicine, Chulalongkorn University, Bangkok, Thailand; 4 Faculty of Geography, Chiang Mai University, Chiang Mai, Thailand; 5 Veterinary Public Health Centre for Asia Pacific and Excellent Center for Veterinary Public Health, Faculty of Veterinary Medicine, Chiang Mai University, Chiang Mai, Thailand

**Keywords:** Spatiotemporal cluster, Model, FMD, Cattle, Northern Thailand

## Abstract

**Background:**

Foot and mouth disease (FMD) is a highly infectious and contagious febrile vesicular disease of cloven-hoofed livestock with high socio-economic consequences globally. In Thailand, FMD is endemic with 183 and 262 outbreaks occurring in the years 2015 and 2016, respectively. In this study, we aimed to assess the spatiotemporal distribution of FMD outbreaks among cattle in Chiang Mai and Lamphun provinces in the northern part of Thailand during the period of 2015–2016. A retrospective space-time scan statistic including a space-time permutation (STP) and the Poisson and Bernoulli models were applied in order to detect areas of high incidence of FMD.

**Results:**

Results have shown that 9 and 8 clusters were identified by the STP model in 2015 and 2016, respectively, whereas 1 and 3 clusters were identified by the Poisson model, and 3 and 4 clusters were detected when the Bernoulli model was applied for the same time period. In 2015, the most likely clusters were observed in Chiang Mai and these had a minimum radius of 1.49 km and a maximum radius of 20 km. Outbreaks were clustered in the period between the months of May and October of 2015. The most likely clusters in 2016 were observed in central Lamphun based on the STP model and in the eastern area of Chiang Mai by the Poisson and Bernoulli models. The cluster size of the STP model (8.51 km) was smaller than those of the Poisson and Bernoulli models (> 20 km). The cluster periods in 2016 were approximately 7 months, while 4 months and 1 month were identified by the Poisson, Bernoulli and STP models respectively.

**Conclusions:**

The application of three models provided more information for FMD outbreak epidemiology. The findings from this study suggest the use of three different space-time scan models for the investigation process of outbreaks along with the follow-up process to identify FMD outbreak clusters. Therefore, active prevention and control strategies should be implemented in the areas that are most susceptible to FMD outbreaks.

## Background

Foot and mouth disease (FMD) is a highly contagious disease of cloven-hoofed animals. It is known to affect livestock and wildlife and is caused by a virus in the family *Picornaviridae*. The FMD virus has seven antigenically distinct serotypes [1–4]. FMD is considered one of the most important livestock diseases in the world [5]. It has been classified as a multiple species disease and has the potential for rapid and extensive spread both within and between countries. It can also cause severe socio-economic impacts and poses a significant threat to public health consequences according to the World Organization for Animal Health [6]. The disease is generally characterized by vesicular lesions and erosions that are mainly found in and around the oral cavity and the interdigital skin of the feet. The severity of the clinical signs can vary with the strain of the virus, the degree of exposure, the age and breed of the animal, the host species and the degree of host immunity. However, mortality is generally low in cloven-hoofed species but is higher in young animals. This is due to certain complications such as myocarditis, which can lead to more severe cases and even death in young animals [3, 7]. The possible incidence of an outbreak is increased where the high density of cattle contributes to the transmission of the virus due to the fact that the FMD virus is highly infectious and can be transmitted by various routes (e.g. direct contact with infected animals, ingestion, inhalation, etc.) [3]. Common strategies being enforced for the prevention and control of FMD include vaccination, quarantine measures and attempts to stamp out the disease [8–10].

Over the last decade, several outbreaks of FMD affecting livestock have been reported across Africa, South America and Asia [3, 11, 12]. Similarly, Thailand has experienced several incursions of FMD outbreaks in livestock within its borders [12]. FMD is regarded as one of the most important transboundary animal diseases in need of control and eradication in the Association of South East Asian Nations (ASEAN). Thailand established a national FMD strategic plan from 2008 to 2015 in accordance with the South-East Asia and China Foot and Mouth Disease (SEACFMD) campaign and its 2020 FMD road map that encouraged vaccination programs [13]. Therefore, the prevention and control of this disease remains a great challenge in geographic areas that are susceptible to epidemic outbreaks of FMD. The implementation of improved control measures of the disease in endemic areas will reduce the risk of outbreaks and help create disease-free locations [4].

A successful approach to FMD epidemiology will involve early detection and immediate responses to active investigations and surveillance programs. Timely information on the spread of cases in space and time can facilitate action by veterinarians and/or public health officials. Consequently, a practical method for specifying geographical areas and periods of time is the method of spatiotemporal cluster analysis [14], which is now commonly used for disease cluster detection in the public health sector [15–18] and in veterinary sciences [19–22].

Scan statistic is a method used in cluster detection that allows researchers to locate the exact cluster in space and/or time that can then be tested for statistical significance. The space-time scan statistic serves as an extension of the purely spatial analysis where scanning cylinders replace circular windows. There are three main space-time scan statistic models that have been used in the last decade including space-time permutation and the Poisson and Bernoulli models. Briefly, the space-time permutation scan statistic estimates the expected disease occurrence using only case data, while the Poisson model uses data on the background of the population at risk and the Bernoulli model requires specific case and control data [23]. SaTScan is the most commonly used form of detection in the cluster technique and has been applied widely within the public health sector and in veterinary sciences [16, 24–27]. Up to now, the implementation of the space-time cluster detection method has been published widely in public health literature, but, utilization of the space-time scan statistics in epidemiology research on FMD has been limited to only a few studies in Iran [28], Mongolia [29], Israel and Palestine [14], and Kazakhstan [30]. FMD cluster identification can highlight changing patterns in risk areas and help to identify new risk areas that can then lead to effective control action plans during the outbreak period, as well as to optimize the preventive measures needed to decrease the degree of disease incursion within the outbreak areas [14].

In Thailand, there is relatively little information available that addresses the application of space-time clustering analysis on the patterns of FMD, which has limited our understanding of the dynamics of the spread of FMD. Notably, there were 183 and 262 outbreaks of the disease across the country in 2015 and 2016 [31]. We selected the provinces of Chiang Mai and Lamphun for the location of this study as they have been identified as endemic areas for the occurrence and spread of FMD in northern Thailand [32].

The aim of the present study is to detect the potential spatiotemporal clusters of FMD outbreaks that occurred among cattle herds in Chiang Mai and Lamphun provinces in the northern part of Thailand in 2015 and 2016 using spatiotemporal models. The detection of clusters and their relative time-frames is crucial in supporting effective FMD management in Chiang Mai and Lamphun; thereby, resulting in the launching of prevention measures that focus on the areas that are most sensitive to FMD outbreaks.

## Results

### Descriptive statistics

**Fig. 1:**
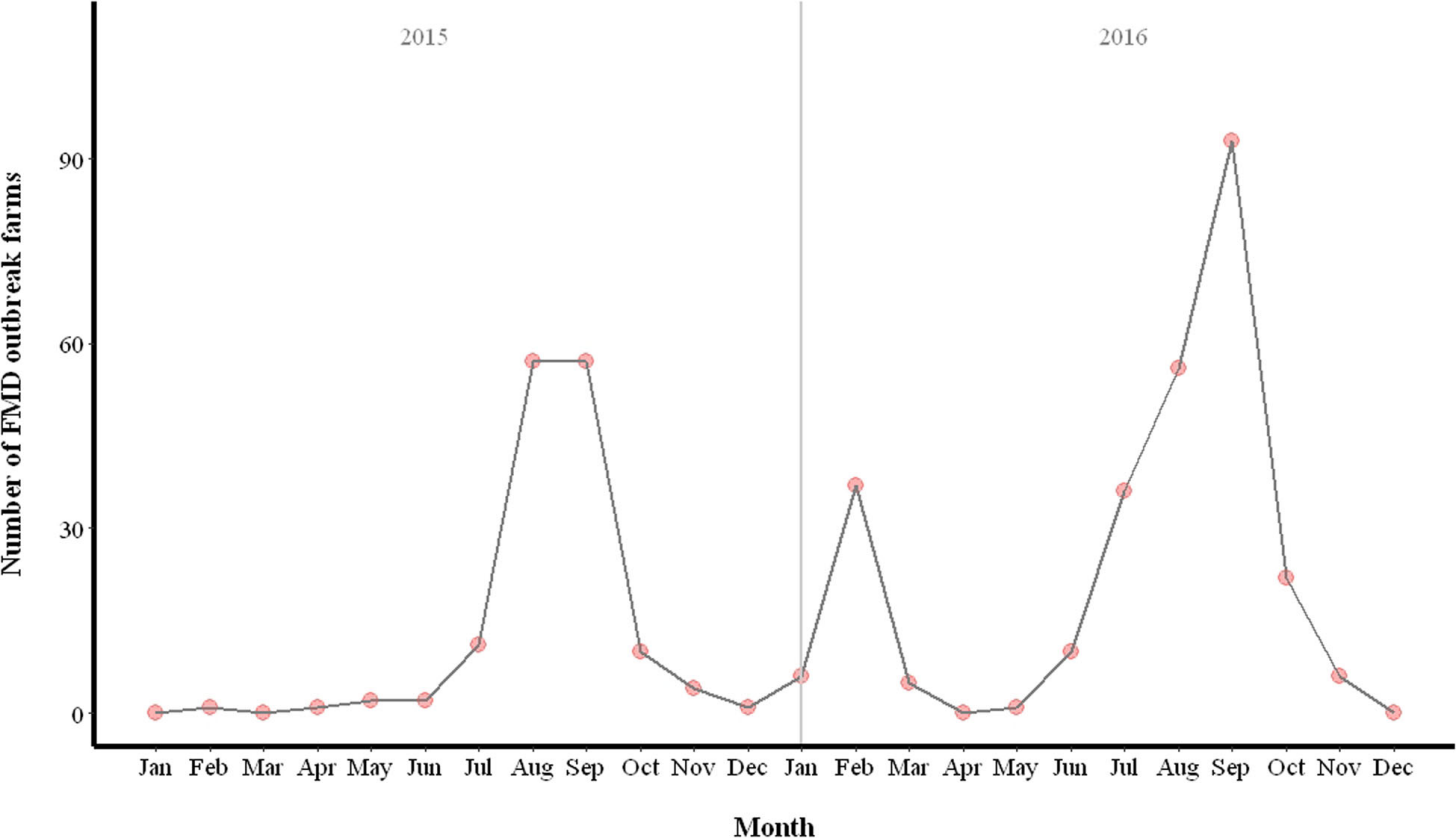
The number of FMD outbreak farms from outbreak investigations data collected by livestock officers in 2015–2016

In brief, FMD outbreaks were found to have occured in 5 districts in Chiang Mai and 5 districts in Lamphun. During the 2015 and 2016 period, 146 and 272 FMD cattle farms were identified as FMD outbreak farms by DLD investigations. Details on the number of FMD and non-FMD outbreak farms by district and year are presented in Table 1. In 2015, the peak of the FMD outbreak occurred from July to October, while in 2016, there were 2 peaks of FMD outbreaks that were recorded from January to March and from June to November (Fig. 1).

**Table 1:** Number of cattle farms and number of FMD outbreak farms for FMD outbreak in Chiang Mai and Lamphun during 2015–2016 by districts

Location*	2015		2016	
	Number of cattle farm	Number of FMD outbreak farm (percentage)	Number of cattle farm	Number of FMD outbreak farm (percentage)
Chiang Mai				
MO	70	23 (32.85)	418	126 (30.14)
SK	35	17 (48.57)	175	42 (24)
SS	136	91 (66.91)	145	30 (20.68)
MR	0	0	3	3 (100)
DS	0	0	2	2 (100)
Lamphun				
BH	4	0	44	2 (4.54)
BT	39	14 (35.89)	61	24 (39.34)
ML	7	0	143	40 (27.97)
PS	0	0	44	2 (4.54)
MT	10	1 (10)	90	1 (1.11)
Total	301	146 (48.50)	1125	272 (21.17)

*District name: MO = Mae On, SK = San Kamphaeng, SS = San Sai, MR = Mae Rim, DS = Doi Saket, BH = Ban Hong, BT = Ban Thi, ML = Muang Lamphun, PS = Pa Sang, MT = Mae Tha

### Cluster analysis

#### Spatiotemporal clusters by STP model

**Fig. 2:**
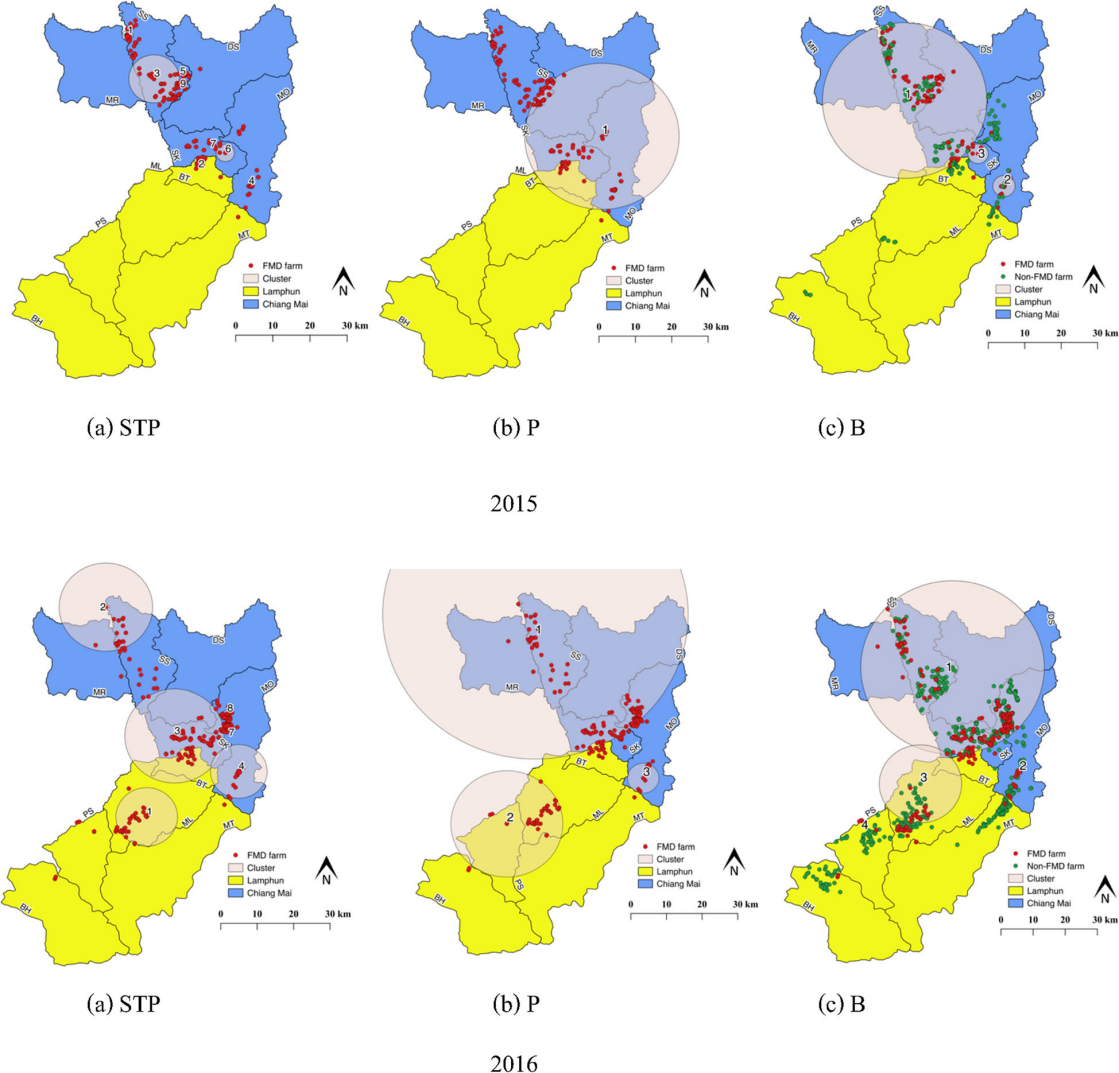
Geographical locations of the clusters of FMD outbreaks in cattle farms in Chiang Mai and Lamphun by **a** space-time permutation model (STP), **b** Poisson model (P) and **c** Bernoulli model (B) in 2015 and 2016, which illustrates locations of FMD outbreak farms (red points) and non-FMD outbreak farms (green points). The map was created using QGIS (version 2.18.28), QGIS Geographic Information System, Open Source Geospatial Foundation Project, all content is licensed under Creative Commons Attribution-ShareAlike 3.0 licence (CC BY-SA), available at (http://qgis.osgeo.org)

The space-time permutation scan statistic test detected nine and eight significant (*p* <  0.001) spatiotemporal clusters of FMD outbreaks in Chiang Mai and Lamphun in 2015 and 2016, respectively (Fig. 2a and Table 2). In 2015, the results showed that the most likely clusters were located primarily in the northern SS district of Chiang Mai (19.017796 N, 98.962637 E) with a radius of 1.49 km. This took place from 14 to 27 August 2015 during which time 12 cattle farms with outbreaks were observed (*p* <  0.001). The second most likely clusters were illustrated in Fig. 2a and indicated the spread of the disease in some areas of Chiang Mai and Lamphun, especially in the northern and western SS, southern MO, central SK and northern BT districts. The cluster radius ranged from 0 to 6.84 km. The cluster time of the second most likely clusters varied from June to November, 2015 and mostly occurred in September 2015 (five clusters). The centroid of the most likely cluster in 2016 was observed in the central area of the ML district of Lamphun (18.525698 N, 99.046928 E), of which the radius was 8.51 km for 22 cattle farms (*p* <  0.001). The cluster time was recorded from 5 June to 16 July 2016. The second most likely clusters were pictured in Fig. 2a in northern SS, SK, southern and western MO and northern BT. The cluster radius ranged from 0 to 13.68 km, and the cluster time varied from January to March and June to October, 2016. Our study observed one larger (2015) and four smaller (2016) spatiotemporal clusters. Most smaller clusters that were revealed by the permutation model were located in the eastern SS district (three clusters) in 2015 and the western MO district (four clusters) in 2016, respectively.

**Table 2:** Spatiotemporal clusters by space-time permutation scan statistic model on FMD outbreaks in cattle farms in Chiang Mai and Lamphun, Thailand 2015–2016

Cluster number	Cluster type	Cluster time	Centroid(X,Y)/ Radius (km)	Number of cases	Number of expected cases	Observed to expected ratio	Log likelihood ratio	*p*-value
2015								
1	Most likely	14–27 Aug	19.017796 N, 98.962637 E/1.49	108	7.59	14.22	189.63	< 0.001
2	Secondary	18–24 Sep	18.697055 N, 99.145108 E/1.12	73	5.70	12.80	120.29	< 0.001
3	Secondary	25 Sep-1 Oct	18.906281 N, 99.030852 E/6.84	168	39.98	4.20	118.73	< 0.001
4	Secondary	4–17 Sep	18.630120 N, 99.274667 E/0.60	131	27.81	4.71	103.42	< 0.001
5	Secondary	30 Oct-5 Nov	18.911784 N, 99.104971 E/0.26	35	0.79	44.29	98.84	< 0.001
6	Secondary	28 Aug-3 Sep	18.715834 N, 99.214166 E/2.50	103	18.61	5.54	94.23	< 0.001
7	Secondary	6–12 Nov	18.725603 N, 99.189108 E/0	30	0.74	40.79	82.26	< 0.001
8	Secondary	26 Jun-2 Jul	18.914357 N, 99.117265 E/0	30	1.12	26.72	69.96	< 0.001
9	Secondary	11–17 Sep	18.880194 N, 99.111250 E/0.26	50	7.87	6.35	50.89	< 0.001
2016								
1	Most likely	5 Jun-16 Jul	18.525698 N, 99.046928 E/8.51	156	13.21	11.81	246.09	< 0.001
2	Secondary	17–23 Jul	19.075278 N, 98.940000 E/12.86	196	24.56	7.98	241.05	< 0.001
3	Secondary	28 Aug-1 Oct	18.737536 N, 99.119652 E/13.68	588	242.21	2.43	200.09	< 0.001
4	Secondary	31 Jan-5 Mar	18.644958 N, 99.288258 E/7.82	200	36.75	5.44	180.49	< 0.001
5	Secondary	14–27 Aug	18.765499 N, 99.261678 E/0.57	66	4.75	13.89	113.08	< 0.001
6	Secondary	17–23 Jan	18.784143 N, 99.255501 E/0	30	0.32	93.77	106.70	< 0.001
7	Secondary	7–13 Feb	18.760247 N, 99.262097 E/0	30	0.37	80.37	102.12	< 0.001
8	Secondary	24 Jul-6 Aug	18.797000 N, 99.258491 E / 1.12	99	25.67	3.86	61.26	< 0.001

#### Spatiotemporal clusters by Poisson model

One and three significant (*p* <  0.001) spatiotemporal clusters of the FMD outbreak in Chiang Mai and Lamphun were identified by Poisson space-time scan statistic model in 2015 and 2016, respectively (Fig. 2b and Table 3). The most likely cluster in 2015 occurred in the DS, SK and MO districts of Chiang Mai and BT of Lamphun, for which the centroid was located at 18.770903 N, 99.248754 E. This cluster included 81 FMD farms that were located within a 21.31 km radius, and data were recorded over the period of a month (4 September to 1 October, 2015). The relative risk of the cluster was recorded at 6.57 (*p* <  0.001). In 2016, the most likely spatiotemporal cluster was classified in several areas of Chiang Mai that included SS, MR, DS, SK and MO districts (19.046783 N, 98.985592 E), for which the radius of the cluster was 42.59 km. The relative risk of the cluster was recorded at 4.22 (*p* <  0.001), and the cluster time lasted from 6 February to 17 September, 2016. Two of the second most likely clusters were located at PS, ML and MT districts of Lamphun and the southern MO district of Chiang Mai (Fig. 2b) with relative risk values of 4.61 and 2.86, respectively. The radius ranged from 15.80 to 4.44 km, and the cluster time periods were from 31 July to 8 August, 2016 and 31 January to 27 February, 2016, respectively (Table 3).

**Table 3:** Spatiotemporal clusters by Poisson scan statistic model on FMD outbreaks in cattle farms in Chiang Mai and Lamphun, Thailand 2015–2016

Cluster number	Cluster type	Cluster time	Centroid(X,Y)/ Radius (km)	Number of cases	Number of expected cases	Observed to expected cases	Relative risk	Log likelihood ratio	*p*-value
2015									
1	Most likely	4 Sep-1 Oct	18.770903 N, 99.248754 E/21.31	504	105.86	4.76	6.57	449.07	< 0.001
2016									
1	Most likely	6 Feb-17 Sep	19.046783 N, 9 8.985592 E/42.95	1414	543.86	2.60	4.22	674.43	< 0.001
2	Secondary	31 Jul-8 Aug	18.486710 N, 98.909121 E/15.80	176	44.44	3.96	4.61	113.85	< 0.001
3	Secondary	31 Jan-27 Feb	18.608944 N, 99.274140 E/4.44	179	64.71	2.77	2.86	70.25	< 0.001

#### Spatiotemporal clusters by Bernoulli model

FMD spatiotemporal clusters were identified using Bernoulli space-time scan statistic method. Three and four significant (*p* <  0.001) clusters of FMD outbreaks were observed in Chiang Mai and Lamphun in 2015 and 2016, respectively (Fig. 2c and Table 4). The centroid of the most likely cluster recorded in 2015 was located in the MR, SS, SK DS, MO districts of Chiang Mai and in the BT and ML districts of Lamphun (18.855897 N, 99.021103 E), where 175 FMD farms were observed with a relative risk value of 3.63. The radius of the cluster was recorded at 22.75 km, and the cluster time lasted from 1 May to 27 August, 2015. The second most likely clusters occurred in areas of southern MO and in the eastern SK districts of Chiang Mai with radius values ranging between 2.85 and 3.09 km, respectively. The relative risk values were similar to those of the most likely clusters at 3.61 and 3.39, and the cluster time lasted from 28 August to 15 October, 2015 and 28 August to 12 November, 2015, respectively. In 2016, the most likely cluster was identified in the SS, DS, SK, MR and MO districts of Chiang Mai (18.920082 N, 99.112156 E), which included 872 FMD farms in a radius of 25.63 km. The relative risk value of the cluster was 4.12, and the cluster time frame was from 10 July to 12 November, 2016. The second most likely clusters were observed in 3 clusters that were located in western BT, northern and central ML, the northern PS districts of Lamphun and also in the southern MO district of Chiang Mai. One larger and two smaller clusters were identified with radius values of 11.52, 0.75 and 0.66 km, respectively. The relative risk of the second most likely clusters was higher than the most likely cluster in a range from 7.48 to 8.06 km. The cluster time varied over three periods as was shown in Table 4.

**Table 4:** Spatiotemporal clusters obtained by Bernoulli scan statistic model on FMD outbreaks in cattle farms in Chiang Mai and Lamphun, Thailand 2015–2016

Cluster number	Cluster type	Cluster time	Centroid(X,Y)/Radius (km)	Number of cases	Number of expected cases	Observed to expected cases	Relative risk	Log likelihood ratio	*p*-value
2015									
1	Most likely	1 May-27 Aug	18.855897 N, 99.021103 E/22.75	472	166.64	2.83	3.63	435.41	< 0.001
2	Secondary	28 Aug-15 Oct	18.630732 N, 99.282214 E/3.09	283	90.38	3.13	3.61	323.63	< 0.001
3	Secondary	28 Aug-12 Nov	18.715834 N, 99.214166 E/2.85	133	41.74	3.19	3.39	158.20	< 0.001
2016									
1	Most likely	10 July-12 Nov	18.920082 N, 99.112156 E/25.63	1777	826.26	2.15	4.12	806.39	< 0.001
2	Secondary	31 Jan-27 Feb	18.641755 N, 99.290282 E/0.75	149	19.38	7.69	8.06	307.38	< 0.001
3	Secondary	5 Ju-6 Aug	18.610597 N, 99.026988 E/11.52	137	19.12	7.16	7.48	247.16	< 0.001
4	Secondary	28 Aug-8 Oct	18.513051 N, 98.870263 E/0.66	85	11.06	7.69	7.89	174.48	< 0.001

#### Comparison between model outputs

**Fig. 3:**
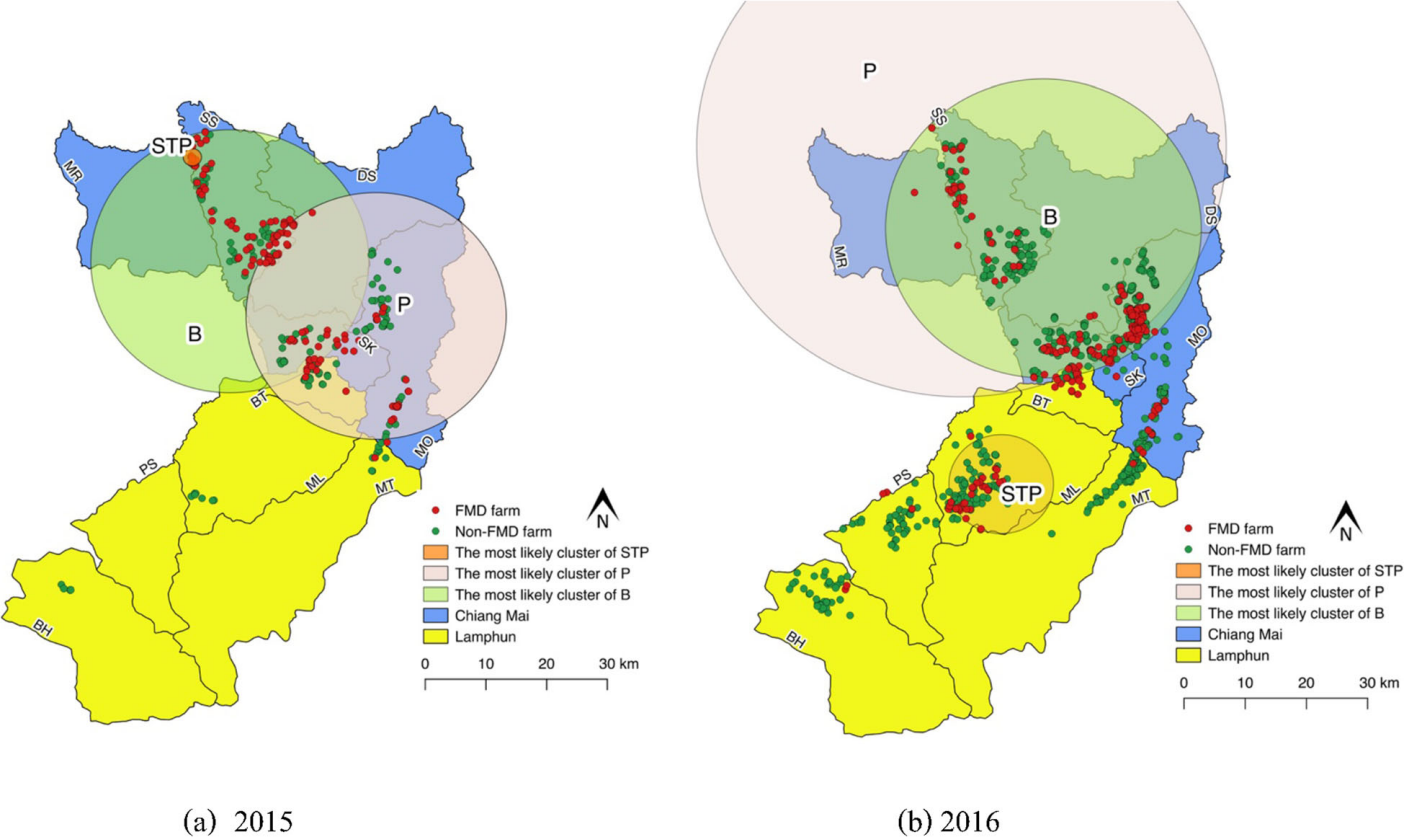
Geographical locations of the most likely clusters of FMD outbreaks in cattle farms in Chiang Mai and Lamphun by space-time permutation model (STP), Poisson model (P) and Bernoulli model (B) in 2015 and 2016. The map was created using QGIS (version 2.18.28), QGIS Geographic Information System, Open Source Geospatial Foundation Project, all content is licensed under Creative Commons Attribution-ShareAlike 3.0 licence (CC BY-SA), available at (http://qgis.osgeo.org)

Some degree of geographical overlapping of the three models occurred (Fig. 3). In 2015, all the most likely clusters were located in Chiang Mai. The entire cluster size of STP was very small (radius 1.49 km) and overlapped with the Bernoulli scanning size, while the Poisson and Bernoulli models had cluster sizes larger than 20 km, for which some had overlapped. All the clusters occurred in the period of the major peak of the FMD outbreak in that year (May to October). The most likely cluster in 2016 was observed in both Chiang Mai and Lamphun provinces. The most likely cluster by STP model was detected separately in Lamphun, whereas the scanning results of the Poisson and Bernoulli models revealed that the clusters were located in Chiang Mai. The cluster size of the STP model (8.51 km) was smaller than that of the Poisson and Bernoulli model (> 20 km). The scanning cluster obtained by the Bernoulli model overlapped with that of the Poisson model. The cluster period obtained from the Poisson model was the longest (approximately 7 months) followed by the Bernoulli model and the STP model, respectively.

## Discussion

This present study is the first report to investigate the spatiotemporal clusters of FMD outbreaks in cattle farms in Thailand. The spatiotemporal scans were conducted in 2015 and 2016 in Chiang Mai and Lamphun provinces using different models incorporating retrospective space-time scan methods that included space-time permutation and the Poisson and Bernoulli models. This was done in order to better understand the cluster patterns. With different models, FMD outbreak clusters were identified. The centers and time frames of the clusters were then found to have either overlapped or displayed no intersection of the geographic areas.

In general, the space-time scan statistics demonstrated several advantages. These included consistent spotting of the cluster geographical areas and cluster times, the trials of statistical significance of the detected clusters and the permutation model that allows for the detection of clusters from case aggregation. Additionally, the Poisson model figures are identified from the populations at risk, while the Bernoulli model requires case and control data. Each model of the space-time cluster analysis in SaTScan reveals potential strengths and weakness. For instance, the space-time permutation scan statistic is suitable for early detection of disease outbreaks, whereas the Poisson model can be applied to anticipate outcomes in order to conduct more suitable models of analyses than the permutation model in cases when the data of the populations at risk are completely accessible [16, 24]. Our study revealed that the most likely clusters and secondary clusters were identified in all models (Fig. 2). The centers and time frames of the clusters showed some areas of overlapping or with no intersection between models. These findings showed that the space-time permutation technique detected more clusters in both 2015 and 2016 than other models resulting in differences in the data obtained from the models. However, the outputs from different models are based on disease outbreak data used for this study. For example, a previous research study showed that the permutation and the Poisson models produced similar numbers and a similar average time period of the clusters for highly pathogenic avian influenza study [27].

Because the FMD outbreak data was available for conducting the STP and the Poisson and Bernoulli models, we analyzed the FMD outbreak data using different models in order to obtain information for FMD surveillance based on space-time analysis. Differences in data and analytical methods provided different results, but useful knowledge was obtained. According to our study, the occurrence of FMD spatiotemporal clusters detected by the space-time permutation model was employed to illustrate the spatial patterns that were mainly situated in the SS, SK, MO districts of Chiang Mai and the BT district of Lamphun in 2015. Subsequently, risk outbreak cluster areas changed to the ML and BT districts of Lamphun and the SS, MR, SK, MO districts of Chiang Mai in 2016. These results suggest that the risk outbreak locations were most commonly found within dairy cattle farming operations. According to our data, the size of the spatial radius of each model varied, for example, the radii of the Poisson results were more likely greater than those of the other methods. The STP model presented a radius that varied from 0 to 13.68 km, whereas, the Poisson and Bernoulli models showed higher radius values at 4 to 42 km and 0.66 to 25.63 km, respectively. However, these results suggest that the widest radius could be used for FMD control in high risk areas, as one study revealed that the largest cluster was more than 30 km for the effective control of an FMD epidemic within the outbreak area [14]. In 2015, the temporal distribution of the clusters identified by STP, Poisson and Bernoulli models suggested that FMD outbreaks were likely to occur during May to November. However, in 2016, outbreak distributions were likely to be observed during January to March and June to November. These findings were consistent with a previous report that showed that the greatest FMD outbreak period took place in Chiang Mai from January to June [32]. One of the possible reasons for this could be related to the intensive contact that resulted from animal movements within live cattle markets that were prevalent during this period of time [32]. It is worth to discuss here regarding to likelihood ratio and relative risk from Bernoulli model. The SaTScan Bernoulli model used a likelihood ratio test of the probability of a group of FMD outbreak farms within a potential cluster defined by a circle being a case versus a control in detecting clusters [24, 33]. A set of clusters will be ordered in descending order by value of their LLR and the highest LLR value was found for the most likely cluster [24] as shown in Table 4. In contrast, the RR is ratio of risk of FMD for cattle farms inside the particular cluster and risk of FMD for cattle farms outside the particular cluster [24]. Therefore, it is possible that the most likely cluster for FMD outbreak in 2016 had a lower RR value compared to secondary clusters.

It is important to discuss about the impact of the differences in number of cattle farms between 2015 and 2016 on spatiotemporal models (Table 1). For STP model, there was no impact of this differences because STP model use only number of FMD outbreak farms. For the Poisson model requiring FMD outbreak farms and cattle farms at risk, the analysis was performed for each year separately, so the shift in farm numbers might not have an impact. Similarly, for Bernoulli model, which requires a number of FMD outbreak farms and a number of cattle farms at risk, although the number of farms at risk is different by the year of outbreak, but we have conducted the model for each year, the effect of the number of farms has been compromised.

The two-year FMD outbreak data demonstrated that the transboundary transmissions associated with FMD outbreaks were distributed across the border from Chiang Mai to Lamphun. The preliminary assessment of this situation might have occurred due to the fact that the operators of some cattle farms located in Chiang Mai and Lamphun did not vaccinate impregnated cattle and this fact consequently played an important role in the situation. Some traders introduced new cattle obtained from other areas into areas populated by local cattle. Many of the local animals had been obtained from other owners without employing policies of isolation and from farmers who had no quarantine system. These occurrences might have led to the common spread of FMD in specific areas. Nevertheless, subsequent studies should be implemented to understand more about the epidemiology of FMD including the risk factors and the dissemination of FMD from one province to another using the statistical epidemiological model or some other form of analysis such as social network analysis that utilizes a mathematical model.

By employing 3 different spatial models in this study, we were able to propose an additional guideline for FMD outbreak investigations for veterinarian authorities. Based on the outbreak investigation procedure [34], there were 3 steps of the investigation that should be employed. Firstly, the investigation must be performed immediately after the outbreak of FMD is observed. STP could be used to present the specific farms that are infected with FMD within the area. Secondly, a follow-up in-depth investigation should be conducted that would be aimed at the process of data collection, which would involve information related to cattle population and farm management. Consequently, the Poisson model could be employed. In addition, blood and fluid specimens would be sampled during this stage. Finally, once the FMD outbreaks were controlled, the investigation was mainly focused on determining the risk factors by comparing the management practices, the biosecurity for FMD and other factors related to FMD outbreak farms and the control farms (non-FMD outbreak farms) through the use of the Bernoulli model.

We have proposed guidelines for FMD investigations for Thailand and other countries that have had similar outbreaks and undergone similar investigations. For the first outbreak investigation, the STP model is suggested because the investigation involved only FMD outbreak farms and not control farms due to concerns over the risk of the disease spreading. Therefore, the STP model provides a quick snapshot of the outbreak that can reveal the distribution of FMD within infected farms in the outbreak area. Once the data on the population of cattle in the farm is collected during the follow-up investigation, the data can be applied to the Poisson modeling. Finally, the Bernoulli model should be employed. The advantage of the Bernoulli model is that researchers are able to investigate the risk factors for each case and those of the control farms that are located within the same clusters. In this study, the clusters that were identified by the STP and Poisson models were not much different and some clusters overlapped (Fig. 2), but this was not always the case because the findings of the clusters depended on the outbreak data that was used. The differences observed in the clustering procedure that were identified by 3 spatial models offer valuable information to veterinary authorities, farmers and other stakeholders with regard to the control of the disease. We have suggested that the field FMD outbreak investigation should incorporate the spatial model, which should be performed for improved understanding of disease outbreaks. Importantly, this may support the prevention and control of FMD in the area. Regarding to the proposed guideline, we suggested that the location of the cattle farms need to be registered and that more information about the farms need to be collected before any outbreak for more effective disease control and disease surveillance planning in the occurrence of an outbreak. In addition, more complete spatiotemporal analysis in detecting disease clusters needs to be performed as early as possible for more effective disease control in the right direction.

Notably, the present study did have some limitations. Firstly, it may have underestimated the number of FMD outbreak farms from the outbreak investigation because farmers might not have informed the local veterinary officers about potentially mild cases of illness of FMD infected cattle. Furthermore, because the definition of non-FMD outbreak farm was based solely on the absence of animals with clinical signs of FMD without any serological surveys on the farm, some non-FMD outbreak farms might not be true non-FMD outbreak farms (false negative). Secondly, the uncertainty of the model outcomes can be enhanced by small adjustments such as those related to the spatial scale and the dimensions of the model parameters because the spatiotemporal scan results are responsive to different versatile variables and the selection of the parameters. Consequently, the scanning cluster results can also vary significantly, depending upon the levels of the spatial scale [35].

## Conclusions

This study highlighted the spatiotemporal clusters of FMD in Chiang Mai and Lamphun in 2015 and 2016. The space-time permutation, and the Poisson and Bernoulli models determined the clusters of the FMD outbreak farms in the study areas. The most likely cluster obtained by the Poisson and Bernoulli models yielded larger radius areas than those of the STP model. The overlapping of the FMD clusters were identified by different spatiotemporal methods. The guidelines for FMD outbreak investigation based on methods used in this study were proposed. This study provided useful information for the prevention and control of FMD outbreaks.

## Methods

### Study area

**Fig. 4:**
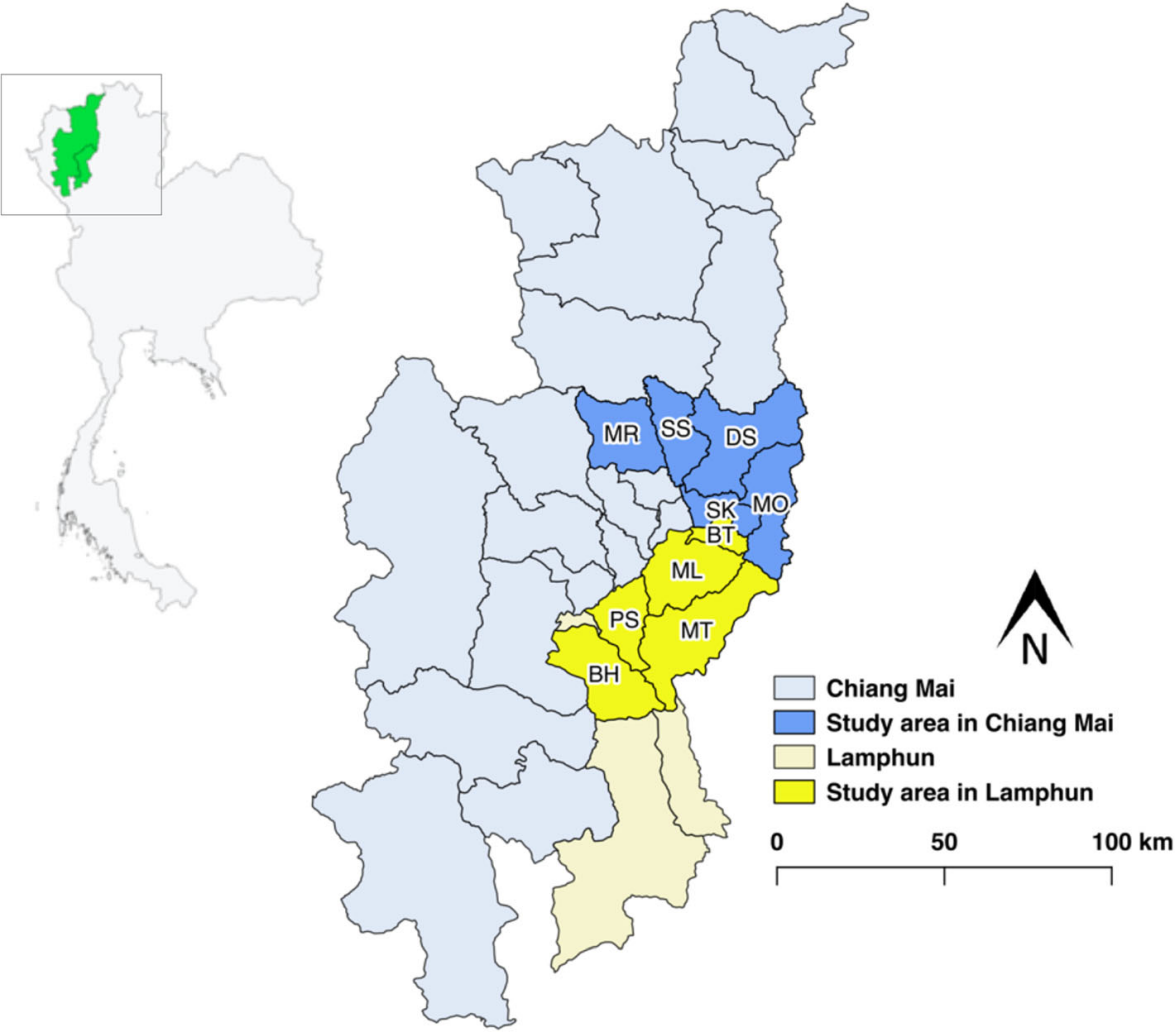
The geographical locations of study in Chiang Mai (blue) and Lamphun (yellow), northern Thailand. The map was created using QGIS (version 2.18.28), QGIS Geographic Information System, Open Source Geospatial Foundation Project, all content is licensed under Creative Commons Attribution-ShareAlike 3.0 licence (CC BY-SA), available at (http://qgis.osgeo.org)

Chiang Mai and Lamphun provinces are located in the northern part of Thailand and are situated at 18.796143 N, 98.979263 E and 18.574462 N, 99.008720 E, respectively (Fig. 4). Chiang Mai and Lamphun cover an area about 20,107 and 4506 km^2^. There are 25 districts and 204 subdistricts in Chiang Mai and 8 districts and 51 subdistricts in Lamphun. The cattle population and household were 195,714 heads and 18,359 households in Chiang Mai and 47,556 heads and 2247 households in Lamphun. The district areas in this study included 5 districts in Chiang Mai including Mae Rim (MR), San Sai (SS), Doi Saket (DS), San Kamphaeng (SK), Mae On (MO) and 5 districts in Lamphun including Ban Thi (BT), Muang Lamphun (ML), Mae Tha (MT), Pa Sang (PS), Ban Hong (BH), respectively. We selected these two provinces because these areas were home to high livestock populations and have experienced a high level of animal movement that could potentially increase the spread of the foot and mouth disease virus [32]. Vaccination programs in these areas were performed three times per year on dairy cattle and two times per year on beef cattle using an inactivated FMD vaccine that was produced by the Department of Livestock Development (DLD).

### Data

FMD outbreak investigative data on cattle of Chiang Mai and Lamphun provinces between 2015 and 2016 were obtained from Chiang Mai and Lamphun Provincial Livestock Offices based on the National Animal Disease Surveillance System (NADSS), DLD. Data consisted of FMD outbreak status, FMD onset outbreak date and geographical location from 1426 cattle farms.

### Unit of analysis and case definition

The FMD outbreak farms were defined as cattle farms in which at least one animal was recorded with typical clinical signs of FMD, including vesicles on the feet, mammary glands, and around the oral cavity by district livestock officers, or tissue samples (e.g. oral epithelium and vesicle lesion tissue) from animal with typical FMD clinical signs were confirmed as being FMDV positive by PCR method, or blood samples from such animals were positive to enzyme-linked immunosorbent assay technique. The laboratory analysis was carried out at the Veterinary Research and Development Center Upper Northern Region, Lampang province, Thailand. The FMD outbreak farms (*n* = 418) were those farms identified as FMD-infected under the Terrestrial Animal Health Code of the Office International des Épizooties [6], for which all models were equally used. Additionally, the non-FMD outbreak farms (*n* = 1008) were utilized for the Bernoulli model. It should be mentioned that the control farms for the Bernoulli model were selected on the basis of the absence of animals with clinical signs of FMD in the farm and no serological surveys have been conducted in those farms.

### Descriptive analysis

The distribution of the investigation of the FMD outbreaks occurred among the cattle farms of Chiang Mai and Lamphun in 2015 and 2016. Data collected at the farm were presented in proportional formats as well as by the time distribution that was utilized to describe the temporal distribution according to month.

### Retrospective space-time scan statistics analysis

The space-time scan statistics are defined by a cylindrical window within a circular geographic base and with a height measurement corresponding to the time of the incident [15, 36, 37]. Briefly, the base and height of the cylindrical windows were varied to identify any potential spatiotemporal clusters. The window was then relocated in space and time based on the specifics of each possible geographical location. The center and radius of the window continuously changed and the height varied according to the likely cluster of time. The scan statistic was adjusted for the uneven geographical density of a target population for all probability models. A likelihood ratio was calculated for each cylinder window. The window with the maximum likelihood represents the most likely cluster.

### Space-time permutation (STP) model

The STP model uses geographical coordinate of FMD outbreak farms to determine clusters of outbreaks. For this technique, a cylindrical space-time window moves across the entire the study area to count number of FMD outbreak farms within the window and estimating the expected number of outbreak farms. The ratio of the observed to expected number of cases and a likelihood ratio was calculated for each cylinder window. Likelihood ratio test was used to evaluate the presence of FMD outbreak clusters. A Monte Carlo simulation (number of replications = 999) was used to determine statistical significance [16]. For this study, the base and height of the cylinder representing to spatial and temporal dimensions were set to contain at most 50% of the reported outbreaks similar to previous reports [15, 38].

### Poisson model

For each period of window scanning, the expected number of cases can be inferred by using the discrete Poisson model with the observed number of cases and the population within and outside the moved windows (potential clusters) of the candidate area during the candidate time. Relative risk (RR) was calculated using the ratio of the observed case number to the expected case number within and outside the window along with the log likelihood ratio (LLR). The *p*-values for the detected cluster were calculated using the Monte Carlo randomization method [16, 39]. The window with the maximum LLR value was defined as the most likely cluster, and the other windows with a statistically significant smaller LLR value were defined as secondary clusters that were then ranked according to their LLR values [18, 40].

Under the Poisson assumption, the LLR for a given window is proportional to:

$$ {\left(\frac{c}{E\left[c\right]}\right)}^c{\left(\frac{C-c}{C-E\left[c\right]}\right)}^{C-c}I\left(\right) $$

where C is the total number of cases, c is the observed number of cases within the scan window, and E(c) is the covariate adjusted expected number of cases within the scan window under the null hypothesis (Ho: spatiotemporal clustering of the study area were obtained by random factors), respectively, C-E(c) is the expected number of cases outside the window, I() is the indicator of function I which is equal to 1 if c > E [c] or 0 otherwise. Since this study was specifically focused on detecting clusters with high rates, I() was set equal to 1 [33].

In this study, the retrospective space-time statistic was used to analyze the spatiotemporal clusters of the FMD outbreak investigation data. A discrete Poisson model was used with longitude and latitude coordinates. The spatial unit was made up of farms, with 418 cattle farms in Chiang Mai and Lamphun; the temporal unit was the day and covered 24 months from 2015 to 2016. The spatial size of the scanning window was limited to 50% of the total population at risk, whilst the temporal size was set to 50% of the total study period in order to scan for any and all small to large clusters. The number of the Monte Carlo randomization model was set at 999. By setting the time frame of the scan analysis to 7 days, we could control the time trends and observe the cluster changes across the entire study period.

### Bernoulli model

The Bernoulli probability model was used to define the aggregation of the FMD outbreak farms around certain focal points. For the Bernoulli model, the likelihood function is determined as follows:

$$ {\left(\frac{c}{n}\right)}^c{\left(\frac{n-c}{n}\right)}^{n-c}{\left(\frac{C-c}{N-n}\right)}^{C-c}{\left(\frac{\left(N-n\right)-\left(C-c\right)}{N-n}\right)}^{\left(N-n\right)-\left(C-c\right)}I\left(\right) $$

where C is the total number of cases, c is the observed number of cases within the window, n is the total number of cases and control within the window, and N is the combined total number of cases and the control within the data set, and I() is the indicator of function I which is equal to 1 if c > C/N or 0 otherwise. Since this analysis is only interested in detecting clusters with higher than expected rates, I() was set equal to 1 [33].

In this study, Bernoulli model was conducted for each year using FMD outbreak farms and non-FMD outbreak farms data to determine clusters of FMD outbreaks. The maximum size of scanning window was defined to include 50% of total farm at risk in each province. The temporal scanning was set as 50% of the reference period for each year (1 January-31 December). The significant of clusters was tested using Monte Carlo simulation method (number of replications = 999) assuming that the FMD outbreak farms were considered as a random sample both in space and time of the total farm population.

Retrospective space-time scan statistical analyses of FMD outbreaks were carried out using SaTScan software version 9.6 [24]. In addition, R statistical software version 3.5.2 with package dplyr and Hmisc (http://www.r-project.org) and QGIS software version 2.18.28 (http://qgis.osgeo.org) were used for the purposes of obtaining data descriptive statistics and map visualization.

## Data Availability

The datasets used and/or analyses during the current study are available from the corresponding author upon reasonable request.

## Abbreviations

ASEAN: The Association of South East Asian Nations
B: Bernoulli model
DLD: Department of Livestock Development
FMD: Foot and mouth disease
LLR: Log likelihood ratio
NADSS: National Animal Disease Surveillance System
P: Poisson model
RR: Relative risk
SEACFMD: The South-East Asia and China Foot and Mouth Disease
STP: Space-time permutation model
